# Molecular Resistance Fingerprint of Pemetrexed and Platinum in a Long-Term Survivor of Mesothelioma

**DOI:** 10.1371/journal.pone.0040521

**Published:** 2012-08-08

**Authors:** Oluf Dimitri Røe, Adam Szulkin, Endre Anderssen, Arnar Flatberg, Helmut Sandeck, Tore Amundsen, Sten Even Erlandsen, Katalin Dobra, Stein Harald Sundstrøm

**Affiliations:** 1 Department of Cancer Research and Molecular Medicine (IKM), Faculty of Medicine, Norwegian University of Science and Technology (NTNU), Trondheim, Norway; 2 Cancer Clinic, Levanger Hospital, Nord-Trøndelag Health Trust, Levanger, Norway; 3 Division of Pathology, Department of Laboratory Medicine, Karolinska Institutet and University Hospital Huddinge, Stockholm, Sweden; 4 Laboratory of Molecular Medical Research, Institute of Clinical Medicine, University of Tromsø, Tromsø, Norway; 5 Department of Pathology and Medical Genetics, St. Olavs Hospital, Trondheim, Norway; 6 Department of Thoracic Medicine, St. Olavs Hospital, Trondheim, Norway; 7 Department of Circulation and Medical Imaging, Faculty of Medicine, Norwegian University of Science and Technology, Trondheim, Norway; Univesity of Texas Southwestern Medical Center at Dallas, United States of America

## Abstract

**Background:**

Pemetrexed, a multi-folate inhibitor combined with a platinum compound is the first-line treatment of malignant mesothelioma, but median survival is still one year. Intrinsic and acquired resistance to pemetrexed is common, but its biological basis is obscure. Here we report for the first time a genome-wide profile of acquired resistance in the tumour from an exceptional case with advanced pleural mesothelioma and almost six years survival after 39 cycles of second-line pemetrexed/carboplatin treatment.

**Methodology and Principal Findings:**

Genome-wide analysis with Illumina BeadChip Kit of 25,000 genes was performed on mRNA from pre-treatment and post-resistance biopsies from this individual as well on case and control samples from our previously published study (in total 17 samples). Cell specific expression of proteins encoded by selected genes were analysed by immunohistochemistry. Serial serum levels of CA125, CYFRA21-1 and SMRP levels were examined. TS protein, the main target of pemetrexed was overexpressed. Proteins and genes related to DNA damage response, elongation and telomere extension and repair related directly and indirectly to platinum resistance were overexpressed, as the CHK1 protein and the genes CHEK2, LIG3, POLD1, POLA2, FANCD2, PRPF19, RECQ5 respectively, the last two not previously described in mesothelioma. We observed a down-regulation of leukocyte transendothelial migration and cell adhesion molecules pathways. Silencing of NT5C in two mesothelioma cell lines did not sensitize the cells to Pemetrexed. Proposed resistance markers are TS, KRT7/ CK7, TYMP/ thymidine phosphorylase and down-regulated SPARCL1 and CDKN1B. Moreover, comparison of the primary expression of the sensitive versus a primary resistant case showed multi-fold overexpressed DNA repair, cell cycle, cytokinesis, and spindle formation in the latter. Serum CA125 and SMRP reflected the clinical and radiological course and tumour burden.

**Conclusions:**

Genome-wide microarray of mesothelioma pre- and post-resistance biopsies indicated a novel resistance signature to pemetrexed/carboplatin that deserve validation in a larger cohort.

## Introduction

Malignant mesothelioma, an aggressive tumour of the pleura and peritoneum, represents a clinical challenge as the incidence increases worldwide, and will continue to increase due to extensive asbestos use in several developing countries [1]. The most effective treatment proven to prolong life of malignant mesothelioma patients is the combination of multi-folate inhibitors, pemetrexed or raltitrexed and cisplatin, but still the median survival is only 12 months, and response rates are approximately 40% [2]. Thus, almost half of the patients are primary resistant and all finally develop resistance. Thymidylate synthase is considered the main target of pemetrexed and current studies indicate that low expression levels of this protein is predictive for good pemetrexed response, longer time to progression and overall survival [3], [4], [5], [6], but the mechanisms and pathways involved in pemetrexed resistance are inadequately elucidated. Several mechanisms of platinum resistance have been described, involving among others the DNA repair system [7], but for mesothelioma treatment still no useful marker has emerged. A resistance profile or signature could have important clinical implications. We recently reported that the gene profile of pleural mesothelioma correlates to several known chemo- and radiation-resistance mechanisms, reflecting the generally resistant mesothelioma phenotype [8]. One of the patients included in our genome-wide expression study responded on pemetrexed and carboplatin for 39 cycles as second-line treatment. At treatment resistance, after 5 years, a new biopsy was obtained from the tumour. Genome-wide profiling was performed as well as immunohistochemistry and serum biomarker expression and the pre- and post-resistance profile was assessed in light of the clinical history. Here we present novel findings and discuss their relevance for mesothelioma treatment resistance and progression.

## Methods

### Ethics statement

The Regional Committee of Research Ethics of Central Norway approved the study protocol and oral and written informed consent was obtained.

### Patient history

**Figure 1:**
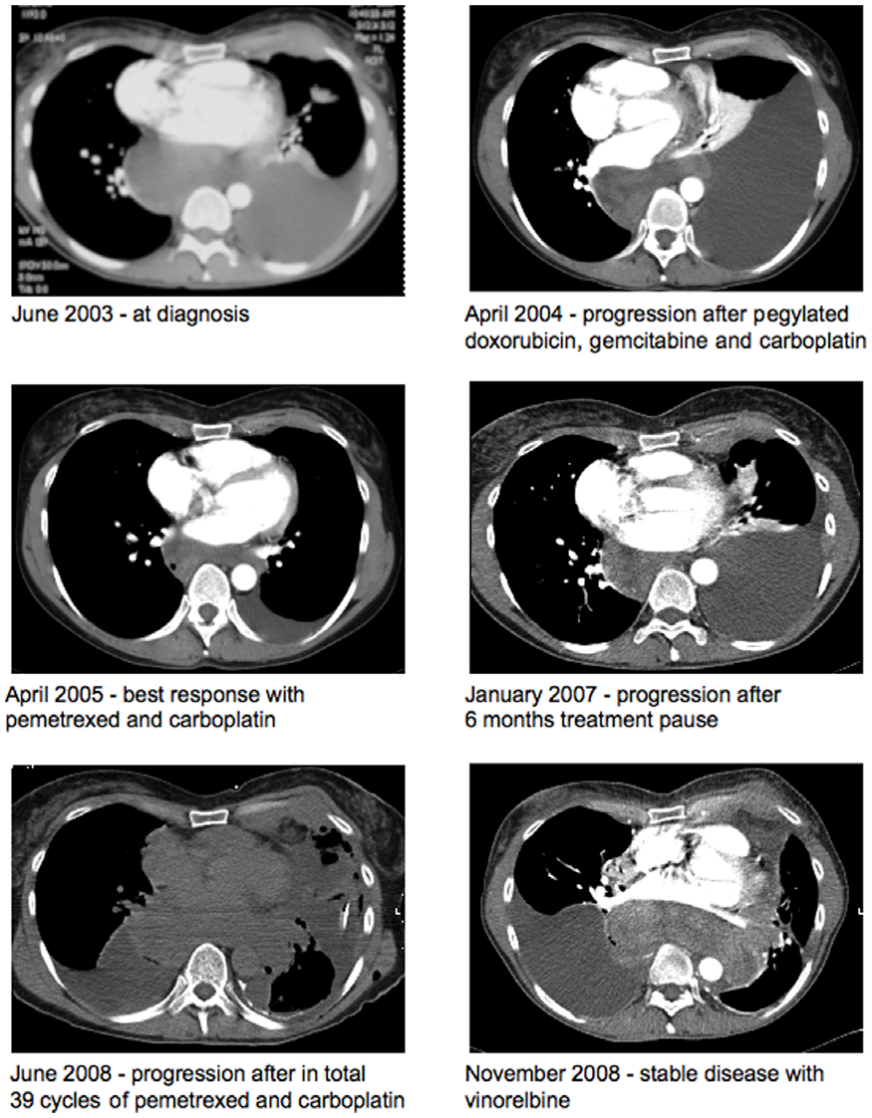
Thoracic computer tomography (CT) throughout the disease course. A large mediastinal tumour as well as thoracic wall infiltration was seen on the left side (CT at the same level). For details see Methods.

A 42-year old woman was referred to our clinic in May 2003 with dyspnoea for the last 8 months, and CT scan showed a large tumour in mediastinum and the left pleura, growing into her left breast (Fig. 1). She had worked nine years as a hairdresser with no obvious asbestos exposure, but the department of occupational medicine discovered that the hair-dryer she had used daily, contained asbestos coils inside. Diagnostic biopsy and fresh frozen material was obtained from pleural tumour by transdermal ultrasound guided biopsy. The histological and immunohistochemical picture showed a clear-cut malignant mesothelioma of the epithelial type (Table 1). Due to her young age, female gender and low-grade asbestos exposure, additional tumour markers were analyzed to exclude other primary solide organ tumours (S-100, Chromogranin, Thyreoglobulin, Calcitonin, TTF-1, Synaptophysin, CK20), but those were all negative. Stage according to the IMIG classification was T4N3M0. As first line therapy, six cycles of carboplatin, pegylated doxorubicin and gemcitabine were given with partial response, but only with slight improvement of clinical status. This was before pemetrexed and platinum was standard treatment. Due to progression almost a year after diagnosis, second line therapy consisting of pemetrexed (500 mg/m^2^) and carboplatin (AUC5) was given every three weeks, with B12 and folate supplement (Fig. 1). Clinical and radiological response was seen after six months, and the condition was considered stable for 24 courses (Fig. 1). After a five months treatment pause, progression was seen and another15 courses of pemetrexed/carboplatin were given, followed by regression, finally stopped due to renewed progression (Fig. 1). In total, she received 39 cycles with minimal toxicity and a self-reported good quality of life. Clinical manifestation of the last progression was dyspnoea and CT scan showed increasing tumour volume and pleural fluid (Fig. 1, June 2008). At that point CT-guided biopsies were obtained for microarray analysis and immunohistochemistry (IHC). Peroral Vinorelbine 80mg/m^2^ at day 1 and 8 was then tried as a third-line treatment with four months of stable disease. The last two months she deteriorated and died at the district hospital. No CT-evaluation or serum samples from that last period were available.

**Table 1:** Change of immunohistochemical expression in tumour before and after resistance.

Markers	Biopsy 2003		Biopsy 2008		
Diagnostic Markers	% positive MM-cells	Intensity (0–4)	% positive MM-cells	Intensity (0–4)	Gene/Protein change*
EMA, cytoplasm	<1	1–2	<1	1	−/−
EMA, cell membrane	40	3	70	3	−/+
Calretinin, nucleus	>95	3–4	>95	4	−/−
mCEA	0	0	0	0	−/−
Ber-Ep4	0	0	0	0	−/−
**Experimental Markers**					
RAD21	>90	3–4	>95	4	−/−
CD138, cell membrane	15	3–4	10	3–4	−/−
CHK1, cytoplasm	<5	1–2	50	1–2	−/+
CHK1, nucleus	<1	1–2	1–2	1–3	−/−
CK7, cytoplasm	20	2–4	95	2–4	+/+
CK7, cell membrane					
Mesothelin	100	3–4	>99	4	−/−
NQO1, cytoplasm	90	1–3	99	1–3	−/+
NQO1, nucleus	20	1–3	70	1–3	
SPARCL, cytoplasm	100	4	100	3–4	--/−
TYMP, cytoplasm	50–60	2–4	85	3–4	+/+
TYMS, cytoplasm	<1	1–2	25	1	+/+
TYMS, nucleus	<1	1–2	<1	1	+/−
VG5Q, cytoplasm	95	2–3	>99	2–3	−/−
VG5Q, nucleus	1–2	1–3	0	0	

* (−) indicates no change, (+) indicate increase and (--) indicate reduction.

Experimental markers are antibodies to proteins encoded by differentially expressed genes in mesothelioma or genes related to mesothelioma biology.

### Biopsies and Microarray analysis

Needle biopsies obtained by CT-guidance were snap frozen for microarray analysis and formalin-fixed for IHC. A high tumour content (>50%) in the samples was morphologically verified by a pathologist (HS). RNA extraction and quality control was done as described previously [9]. In order to maximize reliability of the comparative analysis, we performed microarray analysis of four post-resistance samples (two separate biopsies and two technical duplicates) as well as the mRNA from all mesothelioma and parietal pleural samples described in [9] (in total n = 17). Preparations of 75 ng deep frozen total RNA through first strand cDNA synthesis, second strand synthesis and IVT-reaction to make biotin labeled cRNA was performed using Illumina® TotalPrep(tm)-96 RNA Amplification Kit (Cat#4393543) (Applied Biosystems/Ambion, Austin, TX). cRNA was quantified with a NanoDrop Spectrophotometer (NanoDrop Technologies, Wilmington, DE) and normalized before hybridized to the HumanHT-12 v3 Expression BeadChip Kit (Cat# BD-103-0603) (Illumina, Inc., San Diego, CA) of 25,000 genes. All applications were performed according to the manufacturer's instructions.

After scanning the beads chips the hybridization, biotin labelling, low stringency, housekeeping and negative controls was assessed to determine the quality of technical performance of the bead chips. All control performed as expected (Ref # “Whole -Genome Gene Expression with IntelliHyb Seal – System Manual” – Illumina, 2006). All experiments are registered in ArrayExpress according to the MIAME.

### Microarray statistical analysis

Three separarate analyses were performed; first the tumour versus normal analysis on the Illumina and Affymetrix datasets combined, second on the Illumina dataset aiming at detecting the post-resistance profile, called the acquired resistance analysis, and finally a comparison of the most sensitive versus the most resistant case, using the Affymetrix platform called the intrinsic resistance analysis.

For the tumour versus normal analysis, our previously published dataset on mesothelioma versus normal parietal pleura (n = 16) [8] data with Affymetrix Human Genome U133 Plus 2.0 GeneChip (Accession number EMTAB-47, www.ebi.ac.uk/arrayexpress/experiments/E-MTAB-47) and the dataset with the current Illumina data on the identical samples (Accession number E-MTAB-1109, www.ebi.ac.uk/arrayexpress/experiments/E-MTAB-1109) were the Affymetrix raw probe set intensities were normalised by robust multi array average (RMA) and the Illumina intensities were log2 and quantile transformed. The control of false positives was done according to Benjamini and Hochberg [10], [11] and genes with corrected P-values smaller than 0.05 were taken as significant. The two datasets were separately analysed with the limma package in Bioconductor (www.bioconductor.org). Robust rankings were produced by aggregating results of jackknifed Limma models using the GeneSelector package (www.bioconductor.org). A list of the 1500 top ranked genes from both platforms was chosen (this is an ordered list where a p-value is not available). Moreover, a ranked list of GO terms based on the top 1500 gene-list was produced using Fischers exact test (TopGO software, www.bioconductor.org).

For the aquired resistance analysis, normalization was done as previously described. The data from the primary biopsy was correlated to the averaged data from the biopsies at the time of treatment failure versus the data of the normal parietal pleura samples as described in [8]. The annotation from illuminaHumanv3. db_1.10.0, hgu133plus2. db_2.5.0, org. Hs.eg.db_2.5.0 (www.bioconductor.org) were used. The lists of significant genes were tested for overrepresentation in KEGG PATHWAYS (Kyoto Encyclopedia of Genes and Genomes) [12], and GO (gene ontology) terms [13] using Fishers exact test (significant p<0.05).

For the intrinsic resistance, the pre-resistance gene expressions was compared with the gene expression list of another case in our previously published material with very aggressive disease and innate resistance to radiotherapy and doxorubicin, gemcitabine and carboplatin with only six months survival, using the Affymetrix data [9].

### Immunohistochemistry

Cell specific protein expression encoded by ten mesothelioma-related genes as well as four standard diagnostic antibodies (respective gene symbols in brackets) was assessed by IHC. Calretinin, EMA, CEA and Ber-Ep4 as described in [14] as well as the following, experimental antibodies were tested on formalin fixed paraffin embedded tissues adjacent to samples subjected to microarray: thymidylate synthase (TYMS) (Millipore, Billerica, MA, USA), dilution 1∶50; VG5Q (AGGF1) (Abcam, Cambridge, UK), dilution 1∶500; Chk1 (CHEK1) (Epitomics, CA, USA), dilution 1∶10, overnight incubation at −4°C; NQO1 (NQO1) (Zymed Laboratories, Carlsbad, CA, USA), dilution 1∶50; RAD21 (RAD21) (Abcam, Cambridge, UK), dilution 1∶500; mesothelin (MSLN) (Novocastra Laboratories, Newcastle, UK), dilution 1∶10, overnight incubation at −4°C; thymidine phosphorylase (TYMP/ECGF1) (Sigma-Aldrich, St. Louis, MO, USA), dilution 1∶50; cytokeratin 7 (CK7) (DAKO, Glostrup, DK), dilution 1∶500; syndecan-1 (CD138) (DAKO, Glostrup, DK), dilution 1∶50; hevin (SPARCL1) (R&D Systems, Abingdon, UK), dilution 1∶50. Selected positive and negative controls were included for all antibodies. Slides were reviewed by HS and immunoreactivity was registered as the percentage of stained tumour cells and staining intensity was scored from 1–4 (Table 1).

### Serum biomarker analysis

Blood samples for biomarker analysis were obtained at several consecutive visits in our hospital, and they were immediately centrifuged and stored in −20°C for 1–2 days and subsequently in −80°C. Serum samples were analysed “in batch” for CA125, CYFRA 21–1 and SMRP as described in [15].

### NT5C silencing

Silencing of NT5C was performed with two MM cell lines, the sarcomatoid ZL-34 (kindly provided by Julius Klominek) [16] and the epithelioid M-14-K (kindly provided by K. Linnainmaa) [17]. In our recent experiments the M-14-K cells were sensitive and the ZL-34 cells were resistant to Pemetrexed treatment (unpublished data), making them a suitable pair of cell lines for this study. The cells were cultivated in 90% Gibco RPMI 1640 medium (Invitrogen, Carlsbad, CA, USA) with 25 mM HEPES buffer and 1% L-glutamine (Invitrogen), together with 10% Bovine Serum (Invitrogen). All cells were cultured under 37°C and 5% CO_2_ conditions and grown to confluency in 75 cm^2^ flasks (Sarstedt, Nümbrecht, Germany). Flasks with confluent cells were then trypsinized, spun down and approximately 250 000 cells were seeded in every well of a 6 well plate over night (Nunc, Rochester, New York, USA). The NT5C expression was then silenced according to manufacturer's protocol. Briefly, Lipofectamine^TM^ 2000 (Invitrogen) was mixed in medium with siRNA specific for NT5C (Sigma-Aldrich) (Table S1) or negative control siRNA (MISSION® siRNA Universal Negative Control, Sigma-Aldrich) in siRNA concentration of 40 nM and incubated for 20 min. The seeded cells were then treated with one of the siRNA-lipofectamine complexes for 4–6 hours. Following this, fresh medium and serum was added to the cells and they were grown for additional 18–20 hours before treatment or harvesting the cells for RNA isolation.

### RNA isolation and Quantitative Real Time Polymerase Chain Reaction (qRT-PCR)

The silencing of NT5C was validated by qRT-PCR. Cells were trypsinized, washed in cold PBS and spun down. The RNA was then extracted using the High Pure RNA Isolation Kit (Roche, Mannheim, Germany), according to the manufacturer's protocol. The purity and the yield of the RNA isolations were measured using the NanoDrop Spectrophotometer (Nanodrop Technologies Inc). cDNA were constructed from RNA templates according to the manufacturer's protocol using the First-Strand cDNA Synthesis Kit (GE Healthcare, Little Chalfont, Buckinghamshire, England). In brief RNA was mixed with RNaseOUT^TM^ Recombinant Ribonuclease Inhibitor (Invitrogen), heated and then put on ice. Bulk First-Strand cDNA Reaction Mix, DTT Solution, pd(N)_6_ Primer and the RNA was then mixed and incubate for 1 hour. The purity and concentrations of the cDNA were then measured using the NanoDrop Spectrophotometer. qRT-PCR was performed using the Platinum® SYBR® Green qPCR SuperMix-UDG kit (Invitrogen) according to manufacturer's protocol. The cDNA samples were used in quintuplicate and sense/antisense primers for either NT5C (Cybergene AB, Stockholm, Sweden) or GAPDH [18]. We designed the primers for NT5C using gene sequences from GeneBank (NCBI) (Table S1). The experiments were run in an iCycler machine (Bio-Rad, Hercules, CA, USA), analyzed in Bio-Rad CFX manager^TM^ Software 2.0 and the quantity of NT5C were normalized to the GAPDH reference gene and presented as mean values of at least four independent experiments.

### Cell cycle analysis

After silencing, the cells were given fresh medium and serum and treated with either 90 µM Pemetrexed (Lilly, Indianapolis, IN, USA) or with PBS for the control cells. After 48 hours of treatment the cells were trypsinized and spun down. Cells were then fixated by slowly adding cold ethanol while continuously resuspending the cell pellet. The samples were then stored at 4°C over night. Following this, cells were washed with PBS and mixed with staining solution, containing 50 µg/ml Propidium iodide solution (Sigma-Aldrich) and 100 µg/ml Ribonuclease A (Sigma-Aldrich) and incubated for 30 min at 37°C. The samples were then analyzed using FACSCalibur cytometer (Becton Dickinson, Franklin Lakes, NJ, USA) and CELLQuest pro Software. Cell cycle distribution was evaluated with the Dean-Jett-Fox model using FlowJo 7 (Tree Star Inc., Ashland, OR, USA) for Windows.

### Statistical Analysis

All the results for the ZL-34 and the M-14-K cells are mean values of at least four independent experiments. The error bars represent the 95% confidence intervals and the difference between the mean values of treated and untreated cells or silenced and negative control siRNA cells, were analyzed using student's t-test with one-tailed p-values. Statistical significance was considered at p<0.05.

## Results

### Tumour versus normal analysis

The tumour versus normal analysis as described above showed an almost identical picture of the Affymetrix and Illumina platform as seen in the loading and score-plots and differentially expressed genes were very similar (Figure S1) and there was a 65% overlap of the differentially expressed genes (Figure S2). The top 1500 ranked list and GO entities of differentially expressed genes showed an overexpression of genes involved in mitosis, cell cycle checkpoint and DNA repair and down-regulated multicellular organismal development, inflammatory response and vasculogenesis among others (Table S2 Sheet 1–3).

### Acquired resistance analysis

High quality RNA was successfully extracted from the post-resistance tumour samples and analysed with the Illumina platform as described. The acquired resistance analysis revealed 241 overexpressed and 289 down-regulated genes in the post-resistance samples (Table S2 Sheet 4 and 5). There were 23 significantly overexpressed and 65 down-regulated GO-terms (Table S2 Sheet 6 and 7, p<0.05).

**Figure 2:**
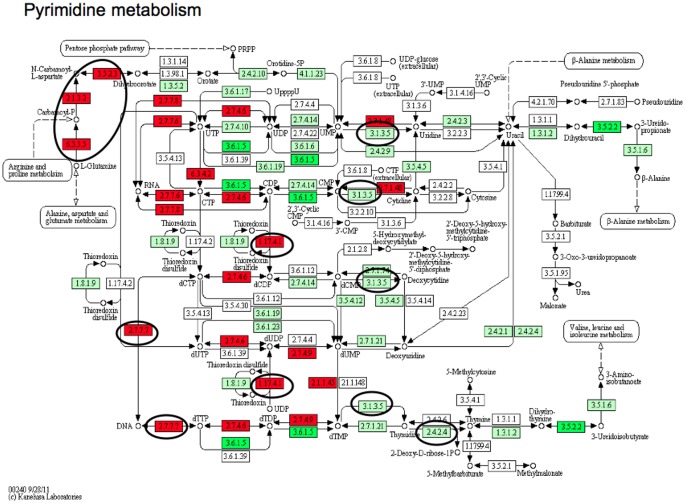
Pyrimidine metabolism in tumour versus normal and at acquired resistance. Genes involved in pathways of DNA metabolism and production in tumour versus normal (red corresponds to overexpressed and dark green correspond to down-regulated) and in acquired resistant tumour (in ovals, all were overexpressed). Abbreviations: 2.1.3.2 =  aspartate transcarbamylase 3.5.2.3 =  dihydroorotase 6.3.5.5 =  CAD; carbamoyl-phosphate synthetase 2 3.1.3.5 =  NT5C; 5′-nucleotidase 2.7.7.7 =  POLA2; DNA polymerase alpha subunit B 2.7.7.7 =  POLD1; DNA polymerase delta subunit 1 1.17.4.1 =  RRM2B; ribonucleoside-diphosphate reductase subunit M2 2.4.2.4 =  TYMP; thymidine phosphorylase.

The largest entity of overexpressed genes in numbers was the metabolic process with 89 genes, whereof 46 genes involved in nucleobase, nucleoside, nucleotide and nucleic acid metabolic process. Other overexpressed entities were tRNA aminoacylation, protein amino acid glycosylation and response to DNA damage stimulus. Similarly in the KEGG (Kyoto Encyclopedia of Genes and Genomes [12]), 15 genes were overexpressed in the metabolic pathways, six and four genes in the pyrimidine (Fig. 2) and purine metabolism respectively and four genes of the aminoacyl t-RNA biosynthesis pathway. Several other important pathways as endocytosis, calcium signalling and ubiquitin mediated proteolysis were also deregulated (Table 2).

**Table 2:** Top 10 overexpressed and down-regulated pathways in post- versus pre-resistance tumour in KEGG pathways map.

Overexpressed genes in KEGG pathways after resistance	
Pathway code and name	Number of genes
hsa01100 Metabolic pathways	18
hsa00240 Pyrimidine metabolism	6
hsa04144 Endocytosis	5
hsa00970 Aminoacyl -tRNA biosynthesis	4
hsa00230 Purine metabolism	4
hsa04020 Calcium signaling pathway	4
hsa04120 Ubiquitin mediated proteolysis	3
hsa05010 Alzheimer's disease	3
hsa04141 Protein processing in endoplasmic reticulum	3
hsa05100 Bacterial invasion of epithelial cells	3

Down-regulated genes in KEGG pathways after resistance	
Pathway code and name	Number of genes
hsa01100 Metabolic pathways	11
hsa04514 Cell adhesion molecules (CAMs)	9
hsa04670 Leukocyte transendothelial migration	9
hsa04530 Tight junction	8
hsa04510 Focal adhesion	6
hsa05200 Pathways in cancer	6
hsa04010 MAPK signaling pathway	6
hsa04060 Cytokine-cytokine receptor interaction	5
hsa00230 Purine metabolism	5
hsa04520 Adherens junction	5

Several interesting and important systems were deregulated after resistance. Note that overexpressed genes in GO metabolic process was 89 and in KEGG only 18. The reason is that KEGG includes new genes in a pathway only when several publications have confirmed it, and thus is more conservative but with a high level of evidence. Most pronounced were the metabolism and modification of DNA and RNA through pyrimidine and purine metabolism and aminoacyl-t-RNA biosynthesis. Interestingly, down-regulation of cell adhesion molecules and leukocyte transendothelial migration as well as cytokine-cytokine receptor interaction was pronounced, also an expression characteristic of mesothelioma versus normal pleura.

The largest entity of down-regulated genes in number of genes was the cellular process with 162 genes. Cell communication and multi-cellular organismal development as well as negative regulation of biological process, transcription and nucleobase, nucleoside, nucleotide and nucleic acid metabolic process were down-regulated. Similarly in KEGG, down-regulation of metabolic pathways, cell adhesion molecules, leukocyte transendothelial migration as well as MAPK-signalling pathway and cytokine-cytokine receptor interaction were the most highly represented pathways.

**Figure 3:**
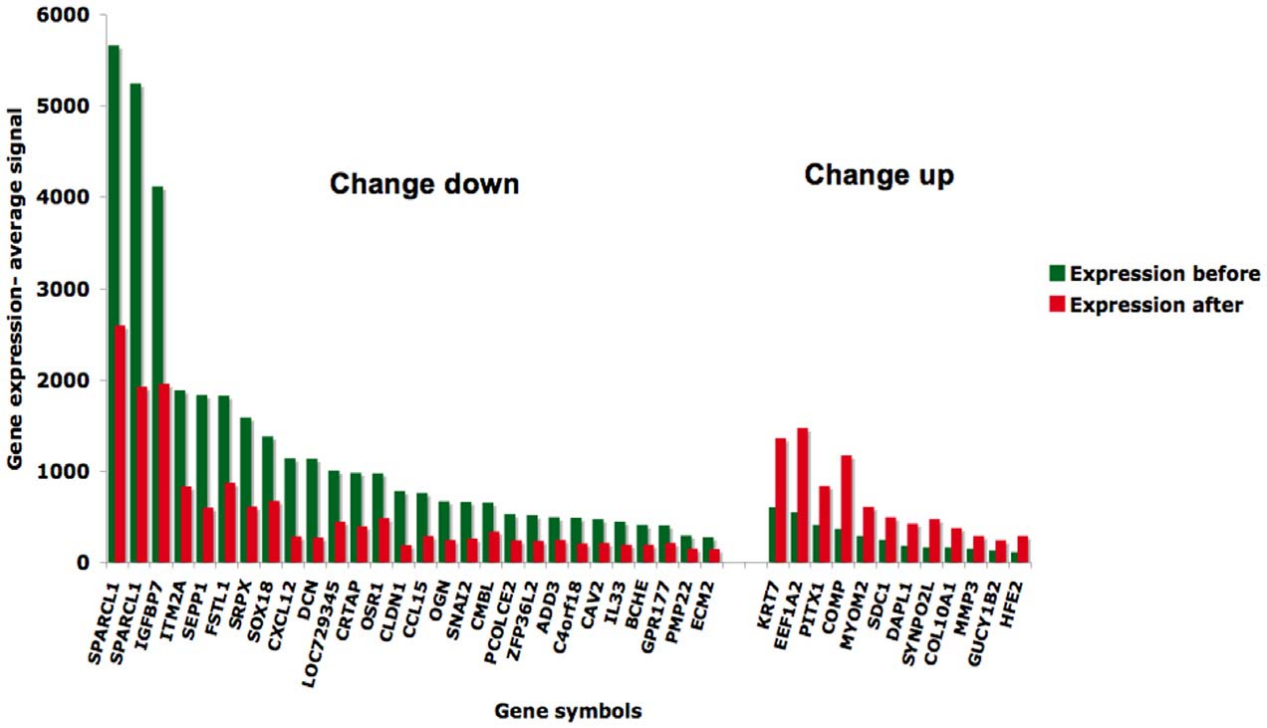
Genes changed more than 2-fold after resistance. Expression of genes that changed more then 2-fold in the post-resistance tumour, compared to initial tumour. Down-regulated genes are shown to the left (30 in total, two not shown) and the overexpressed to the right (15, three not shown). Green bars represent the relative gene expression before and red bars after acquired resistance.

Among the differentially expressed acquired resistance genes, 12 overexpressed and 26 down-regulated genes changed more than 2-fold (Fig. 3).

For examining the relation of acquired resistance genes versus the general mesothelioma gene signature published in [8], the 1500 top ranked genes were analysed for similarities and we found 150 tumour-specific genes were in common (Table S2 Sheet 8).

### Protein expression- IHC

**Figure 4:**
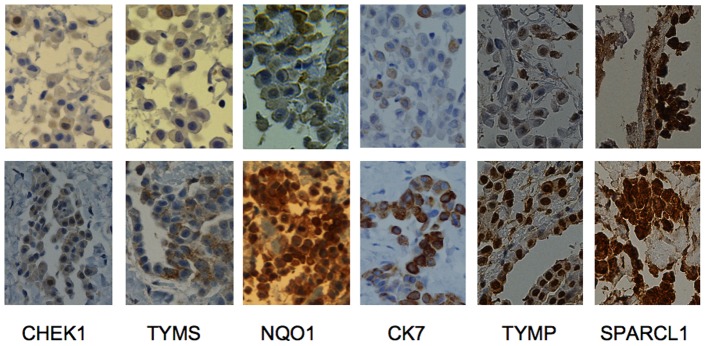
Tumour before resistance and at acquired resistance. Immunohistochemical images (x40) seen before treatment (top row) and five years later after acquired resistance (bottom row). Three of the experimental markers that were hypothesised to be important for mesothelioma aggressiveness as well as resistance, showed increased protein staining after acquired resistance. Chk1 staining increased from below 5% to 50% of the cells, TYMS staining from below 1% to 25% of the cells while nuclear staining of NQO1 increased from 20 to 70% of the cells. Two novel putative resistance markers, KRT7/CK7 and TYMP both gene and protein was significantly increased at resistance. SPARCL1 showed a significant decrease in gene expression, but its encoded protein, hevin, showed only a slight decrease in protein staining intensity.

Due to very limited material for immunohistochemistry (needle biopsy), a careful selection of antibodies was chosen. The histological picture and routine IHC remained virtually unchanged before and after resistance, except for cell membrane EMA staining where immunoreactivity (IR) increased from 40% to 70% of cancer cells (Table 1). Then, experimental markers important of aggressiveness as well as resistance in several cancers as well as mesothelioma, were tested [8]. Damage response protein CHK1 changed from almost no detectable staining in the primary biopsy to 50%, nuclear staining of NQO1 increased from 20 to 70% and TYMS staining increased from below 1% to 25% of tumour cells (Table 1, Fig. 4). RAD21 and mesothelin IHC was analysed for the same reason but showed no change of gene or protein staining.

We previously identified VG5Q as a novel angiogenic overexpressed in mesothelioma [8], but no change was seen. SDC gene was overexpressed, but its protein Syndecan-1 (CD138) an important protein related to the mesothelioma phenotype did not change either (Table 1). KRT7/CK7 and TYMP both gene and protein was significantly overexpressed at resistance (Table 1, Fig. 4). The protein encoded by the down-regulated gene SPARCL1 was only slightly down-regulated after resistance.

### Intrinsic resistance analysis

**Figure 5:**
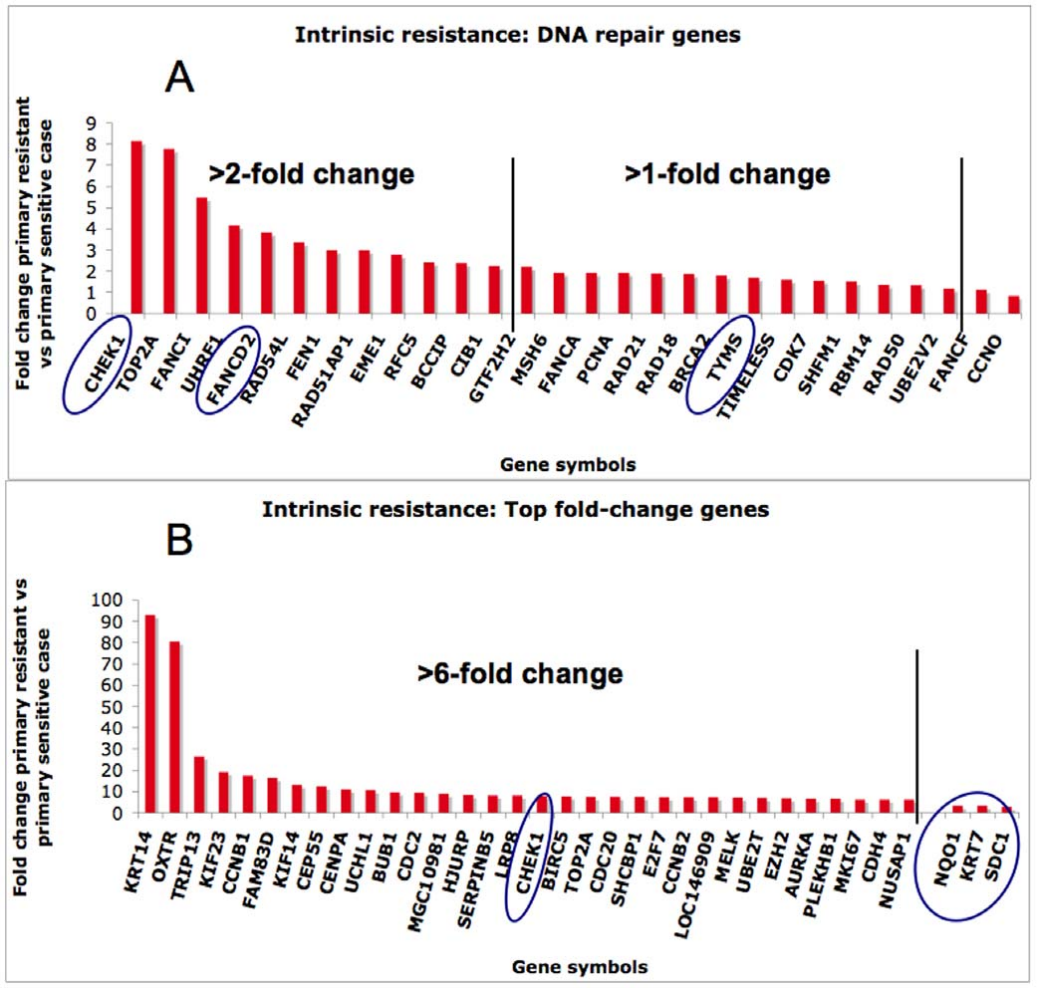
Fold-change of gene expression between a primary resistant mesothelioma with only six months survival and the primary sensitive case with almost six years survival. The top figure shows the DNA repair genes found in [7], depicting a grave overexpression of these genes in the primary resistant case. Among them, CHEK1, FANCD2 and TYMS also seem to be important for acquired resistance (in ovals). Among the 32 top overexpressed genes (arbitrarily >6-fold), the 23 are involved in cell cycle, cytokinesis, and spindle formation, and several are known to be negative prognostic factors in other cancers. The marked differences in indicate which gene functions may be important for aggressiveness and intrinsic treatment resistance in mesothelioma. KRT7 and SDC1 genes that changed significally at acquired resistance were also >2-fold overexpressed in the aggressive case. NQO1, a putative treatment target where protein expression was increased in acquired resistance, was >2-fold overexpressed in the aggressive case.

A simple quantification of gene expression in the long-term survivor at diagnosis and the primary resistant and most aggressive case with only six months survival (raw data from [8]) was arranged by fold change (Fig. 5). Of 828 differentially overexpressed genes, 188 (22.7%) were more than 2-fold overexpressed in the resistant case and only 22 (2.6%) in the sensitive case. Among those, the DNA repair genes and the genes with the highest fold value are discussed. The findings are presented in the respective section.

### NT5C silencing in cell lines

The NT5C expression was silenced to 34±7% of the original expression level in the M-14-K cells and to 23±14% in the ZL-34 cells, respectively. When comparing the mean values of controls cells (for both M-14-K and ZL-34) with siRNA specific for NT5C or negative control siRNA, no significant differences were found when looking on the total amount of cells and on the cell cycle distribution. No significant differences were seen when comparing the pemetrexed treated cells, with silenced NT5C or with negative control siRNA (Figure S3). However, we could see some minor differences between the two groups of Pemetrexed treated ZL-34 cells. After treatment, there was 5 percentage points more live cells in the ZL-34 that had been silenced than in the control cells. The cell cycle distribution was also slightly changed in the silenced cells having 6 percentage points more cells in G1 phase, 9 percentage points less cells in S phase and 5 percentage points more cells in the G2 phase.

### Serum Biomarkers

**Figure 6:**
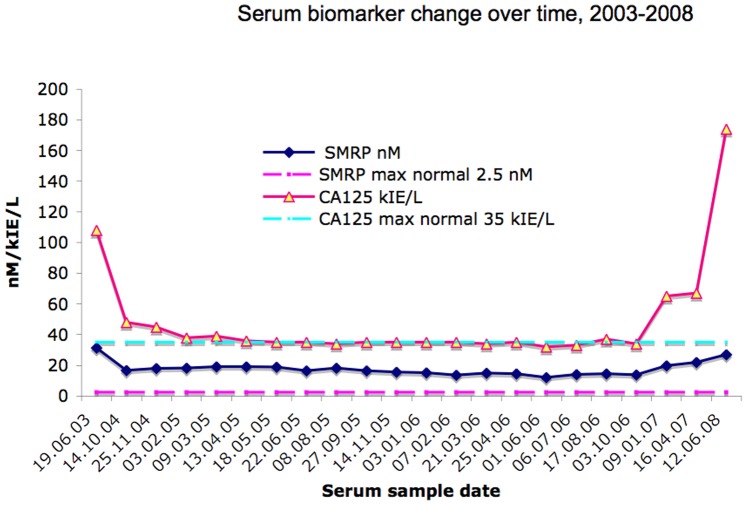
Serum biomarker changes over time. Ca125 and Mesothelin (SMRP) biomarkers in serum were elevated at diagnosis and decreased and increased according to the clinical and radiological regression and progression respectively. The straight horizontal dashed lines depict the maximum normal values of the two markers. CYFRA 21-1 is not shown.

Serum Mesothelin Related Protein (SMRP) was measured at several time-points, as well as CA125 and CYFRA 21-1. CYFRA 21-1 was below the cut-off at diagnosis, and the value increased 2-fold above cut-off only at resistance (not shown). SMRP was 31.33 nM initially, 12-fold the normal value (2.5 nM), decreased to 16.7 nM and was relatively stable until progression after treatment pause, and subsequently increased after resistance to treatment (Fig. 6). CA125 followed a similar course, initially 108 kIE/L, 3-fold normal value of 35 kIE/L, stabilized on 35 kIE/L and increased after treatment pause and thereafter at resistance.

## Discussion

**Figure 7:**
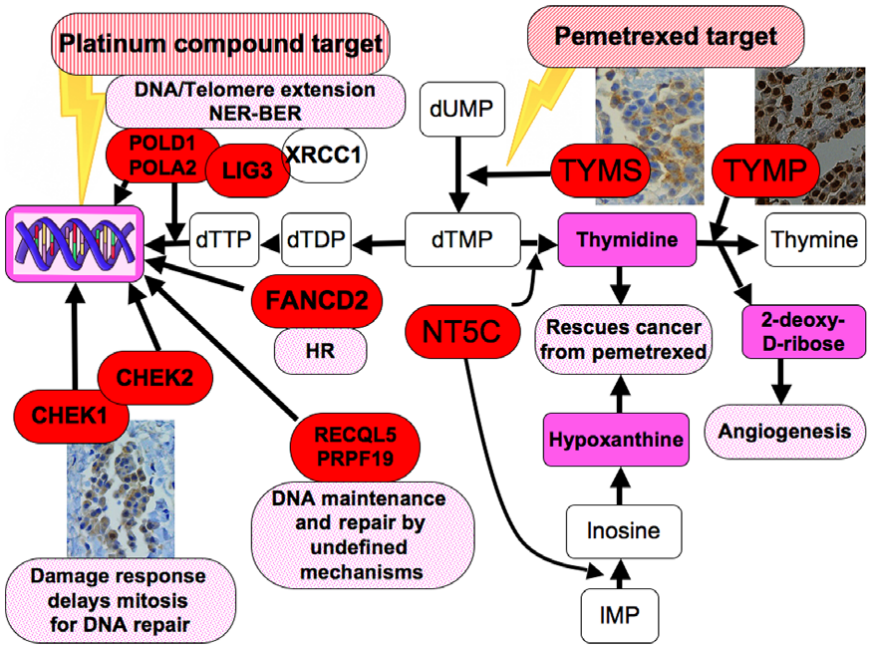
Cartoon summarizing the most important findings related to pemetrexed-platinum resistance and tumour aggressiveness in post- versus pre-treatment biopsies. All genes labelled red are significantly overexpressed except TYMS and CHEK1 where only the encoded protein overexpression was seen. Thymidylate synthase protein overexpression is a known resistance factor against pemetrexed and the TYMP gene/protein was overexpressed. Pemetrexed inhibits the folate enzymes TYMS, GARFT and DHFR. Overproduction of thymidylate and hypoxanthine can reduce the pemetrexed effect on all three enzymes and rescue tumour cells from pemetrexed toxicity. NT5C was overexpressed and encodes an enzyme, 5′, 3′-nucleotidase, a key enzyme for production of thymidylate and hypoxanthine. TYMS and the downstream metabolite 2-deoxy-D-ribose increase angiogenesis and tumour aggressiveness. POLA2 and POLD1 are important for DNA elongation, telomerase extension and cell survival, but also for repair, namely nucleotide excision repair (NER, POLD1 combined with LIG3) and base excision repair (BER, LIG3 with XRCC1) thus important for platinum resistance. Two novel DNA repair genes with undefined mechanism related to both drug and radiation resistance, RECQL5 PRPF19, were overexpressed. Damage response gene CHEK2 and Chk1 protein was overexpressed, both involved in delaying mitosis and facilitating DNA repair. Abbrevations: CHEK1/Chk1; checkpoint 1 kinase CHEK2; checkpoint 2 kinase dTDP; deoxythymidine di-phosphate dTTP; deoxythymidine tri-phosphate dUMP; deoxyuridylate IMP; inositol mono-phosphate LIG3; Ligase III NT5C; 5′, 3′-nucleotidase, cytosolic POLA2; DNA polymerase alpha subunit B POLD1; DNA polymerase delta subunit 1 TYMP; thymidine phosphorylase TYMS; thymidylate synthase.

To our knowledge this is the first published case of malignant mesothelioma with a comparison of genome-wide expression at the time of diagnosis and after pemetrexed-platinum therapy failure. The shortcoming of this study is obviously the one case, along with the caveats of microarray analysis of heterogeneous tumour samples that shows the expression of a tumour/stroma system rather than tumour cells only. Furthermore, the biological material was scarce and thus only selected IHC was used for validation. However, all steps were optimized, from the way the biopsies were obtained (needle biopsy) and handled (rapid freeze and storage), as well as optimal RNA extraction and verification of a high tumour content in all the samples in adjacent biopsy. To improve validity the gene expression of profile after resistance was tested against six mesothelioma and seven normal samples as well as showing overlapping results in two different microarray platforms. Here several important genes and pathways that may reflect tumour response and subsequent resistance were detected. In the following sections results that may have relevance in pemetrexed- and platinum-resistance will be discussed and the main findings are summarized in Fig. 7.

### Metabolic process- DNA and RNA metabolism

The metabolic process entity included several overexpressed genes involved in DNA and RNA metabolism. In pyrimidine metabolism six genes (CAD, NT5C, POLA2, POLD1, RRM2B, TYMP) and four in purine metabolism (NT5C, POLA2, POLD1, RRM2B) were also overexpressed in the KEGG pathways. Pemetrexed is a multifolate antagonist that inhibits replication through folate-dependent enzymes, thymidylate synthase (TS), GARFT and DHFR where the affinity for the TS is many orders of magnitude higher than the latter two, and is recognised as the main target of pemetrexed in cell line experiments [19]. TS encoded by TYMS, is a key protein that catalyzes the methylation of deoxyuridylate (dUMP) to deoxythymidylate (dTMP) that maintains the dTMP pool critical for DNA replication and repair. Low TS expression increases the pemetrexed response in vitro [20], [21]. In our previous study, we noted that this good responder had a very low TYMS mRNA and TS protein expression seen by IHC in contrast to all the other cases with a mean survival of only 12 months [8]. After resistance, TS staining increased from below 1% to 25% of the cells (Table 1, Fig 4) while TYMS mRNA did not change. Interestingly, studies have also shown that the thymidylate synthase protein, and not the gene, is the only marker to predict pemetrexed resistance in mesothelioma, in line with our findings [3], [4], [5].

Acquired pemetrexed resistance of cancer cells *in vitro* and in a murine model *in vivo* has been shown with addition of thymidine that blocks pemetrexed effect on TS, hypoxanthine that blocks GARFT and the combination of thymidine and hypoxanthine that blocks DHFR [20], [21]. In a one-patient study thymidine was successfully used to reverse kidney failure due to pemetrexed, indicating that at least the toxic effects of pemetrexed can be blocked in humans by thymidine [22]. We found overexpression of NT5C, encoding pyrimidine 5-prime nucleotidase also called uridine 5-prime monophosphate hydrolase (UMPH), essential for the catalyzing the dephosphorylation of thymidylate (TMP) to thymidine and inositol monophosphate (IMP) to inositol, the precursor of hypoxanthine. Knockdown of NT5C in colorectal cancer cell lines sensitized cells to 5-FU, a drug also targeting TS [23]. Thus, as elevated levels of thymidine and hypoxanthine have been shown to reverse the pemetrexed effect, an elevated NT5C could thus also be a potential pemetrexed resistance mechanism in vivo (Fig. 7). Because of this, we silenced NT5C in two mesothelioma cell lines, one sarcomatous and one of epithelial type, that were treated with pemetrexed. There was no sensitization of the silenced cells to pemetrexed, only a slight change in cell cycle distribution was seen with in the G1 and G2 phase (Figure S3). Thus, a key role of this gene in pemetrexed resistance could not be determined in vitro for these cell lines. Contributing factors could be that the halftime and activity of NT5C is not known and the actual amount of the protein in the silenced cells is also unknown and this might affect the outcome of these experiments.

Thymidylate phosphorylase mRNA (TYMP) as well as its encoded protein (ECGF1 antibody) was overexpressed at resistance (Fig. 4). TYMP is expressed at higher levels in a wide variety of solid tumours than in adjacent non-neoplastic tissue and been related to tumour progression and aggressiveness. Tumour microenvironment (hypoxia, acidosis) regulates the expression of TYMP, and its expression in tumour tissue shows significant correlation with microvessel density and poor prognosis [24]. TYMP facilitates the conversion of thymidine to thymine and 2-Deoxy-D-ribose, that has been shown as a key control of angiogenesis and tumour progression (Fig. 2) [25]. TYMP is also involved in fluorouracil metabolism and indispensable for the action of capecitabine. Here, we found a low gene and protein expression in the primary setting and highly expressed gene and protein in the post-resistance sample, indicating a role in progression, possibly also in resistance. RRM2B is a ribonucleotide reductase that contributes to DNA repair by supplying deoxynucleotide triphosphate pools in response to DNA damage, and has been associated to treatment sensitivity and tumour invasiveness. Silencing of this gene in prostate cancer sensitizes the tumour cells to both irradiation and doxorubicin [26] as well as reversal of acquired resistance to gemcitabine, which also showed cross-resistance to pemetrexed in cell lines with acquired resistance [27], [28]. RRM2B role in mesothelioma is unknown, but a role in pemetrexed resistance is highly plausible regarding its role upstream of TS and a central role in DNA metabolism (Fig. 2). CAD, encoding a multifunctional protein that initiates and regulates mammalian de novo pyrimidine biosynthesis and is required for mammalian cells to proliferate, was found highly overexpressed. In k-FGF transfected cancer cells that developed resistance to methotrexate, CAD and DHFR were the main genes responsible for the resistance [29]. Furthermore, four genes encoding proteins that load amino acids on t-RNA, MARS, FARS2, WARS, AARS were in the top GO overexpressed group fold-wise, as well as in KEGG pathways. Interestingly, as we have proposed previously, t-RNA aminoacylation could be involved in treatment resistance to ranpirnase [8], [30].

TIMELESS, a circadian rhythm gene involved in DNA damage response and replication was overexpressed in both our studies. Elevated mRNA levels in breast cancer of this positive circadian regulator has been significantly associated with shorter relapse-free survival and recently been regarded as a promising marker of tamoxifen resistance in women with estrogen receptor alpha-positive breast tumours [31].

TNKSBP1, Tankyrase-1 was overexpressed and was previously found to polymerize of poly(ADP-ribose) and to be required for spindle structure and function [32]. TNKSBP1 mRNA in urine sediment from patients with bladder carcinoma correlated with tumour stage, and higher preoperative levels were associated with increased risk of early recurrence [33]. The poly (ADP-ribose) polymerization genes PARP9, 10 and 14 were overexpressed. Generally little is known about their function, but PARP9 and 14 are also called B aggressive lymphoma proteins (BAL-family) and mediates protection against apoptosis at DNA damage [34].

RBBP7, retinoblastoma protein 7, also overexpressed, is one among several proteins that binds directly to the retinoblastoma protein, which regulates cell proliferation. Both mRNA and protein levels were found significantly overexpressed in non-small cell lung cancer tissues and elevated serum levels were highly correlated with distant metastasis [35]. It also plays an important role in epithelial-mesenchymal transition [36]. In general, those genes may play a role in pemetrexed resistance, but less likely in resistance against platinum compounds.

### DNA repair gene overexpression

DNA is the main cytotoxic target of cisplatin and carboplatin by the induction of single and double-strand DNA breaks through adducts and cross-linking, leading to cell death through apoptosis [7]. To counteract the DNA damage induced by a platinum compound, a highly complex repair cascade of several mechanisms is recquired. Recently, the Fanconi anemia/BRCA2 (FA) pathway and Homologous Recombination (HR), a DNA double strand break (DSB) repair mechanism of perfect repair, has been attributed the role as a coordinator of this cascade [37]. In our previous study, several genes involved in HR were overexpressed [8]. Of those genes, only the FANCD2 was overexpressed here. FANCD2 is a key protein in this pathway as it interacts with BRCA2 and further with the members of the FANC and RAD family [37], and could thus play a role in platinum resistance in our case. POLD1 encodes DNA polymerase delta that plays a central role in chromosomal DNA replication, repair, and recombination [38]. It was recently shown to be very important for Nucleotide Excision Repair (NER) [39], known to be important for cisplatin-induced damage (Fig. 7). LIG3 encode DNA ligase III that is a key protein in Base Excision Repair (BER) together with XRCC1, but was also recently involved in NER with POLD1, but also with XRCC1 [40], [41], [42]. LIG3 overexpression was found to predict progression of non-muscle invasive bladder cancer in a large microarray study [43]. Homozygous mutation of LIG3 confers a high risk for pancreatic and other forms of cancer [44].

Some interesting novel DNA repair genes appeared overexpressed in the tumour after resistance. PRPF19 encodes the hPso4 protein that was induced 15- to 30-fold in cells by gamma radiation and chemical mutagens. Loss of hPso4 expression induced by siRNA results in accumulation of double strand breaks, apoptosis, and decreased cell survival after DNA damage and plays a major but previously undefined role in mammalian DNA DSB repair [45]. RECQL5 encodes a helicase, Recql5 that plays an important role in maintaining active DNA replication. It prevents the collapse of replication forks and accumulation of DSB and subsequent cell death after topoisomerase I poisoning by irinotecan, probably acting as a regulator of HR and was recently proposed as a treatment target [46]. Interestingly, Recql5 protein is not widely expressed in normal or non-mesothelioma tumour tissues (www.proteinatlas.org), and may thus be important both for mesothelioma biology and resistance against DNA damaging agents.

Damage response proteins delay the entrance of the damaged cell into mitosis thus facilitating DNA repair. Here the CHEK2 gene was overexpressed (not enough tissue to examine protein expression), and the Chk1 (checkpoint kinase 1) protein, encoded by the CHEK1 gene increased its staining from below 5% to 50% of the tumour cells after resistance (Fig. 4). Chk1 is a putative treatment target, as selectively localized in tumour cells, and is a key protein controlling the G2/M checkpoint and DNA repair as well as playing a role in radiation- and chemo-resistance [47]. Recently, high-throughput RNAi screen identified CHEK1 as target for sensitizing ovarian cancer cells to cisplatin and pancreatic cancer cells to gemcitabine [48], [49] as well as mesothelioma cells to doxorubicin [50]. In our previous study, comparing this case with the most aggressive, the last had an 8-fold higher CHEK1mRNA expression (Fig. 5). Another indication on the relation between CHK1 and pemetrexed is the finding that caffeine, a CHK1 inhibitor sensitizes mesothelioma cell-lines for pemetrexed, and thus may be a putative co-drug target for mesothelioma [51]. CHEK1 appears as a very important gene in resistance towards several DNA-damaging agents, and could be a promising marker for pemetrexed-platinum response and survival in mesothelioma.

### Cell adhesion molecules and leukocyte transendothelial migration

**Figure 8:**
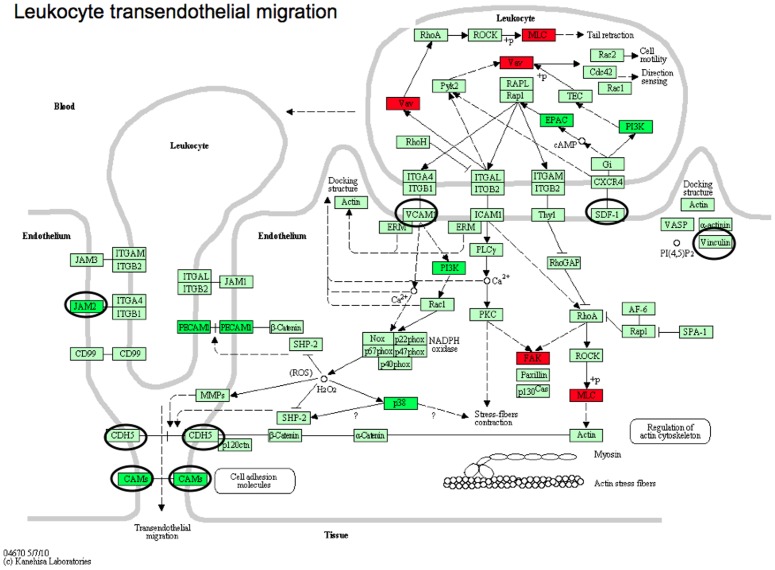
Leukocyte transendothelial migration in tumour versus normal parietal pleura and at acquired resistance. Leukocyte transendothelial migration were among the top down-regulated pathways. Here we show the tumour versus normal profile with overexpressed (red) and down-regulated (green) genes, and genes marked with oval were down-regulated post-resistance. Abbrevations: CDH5; cadherin 5, type 2 (vascular endothelium) CLDN1; claudin 1 CLDN5; claudin 5 CXCL12; chemokine (C-X-C motif) ligand 12 ESAM; endothelial cell adhesion molecule JAM2; junctional adhesion molecule 2 OCLN; occludin (EC:2.1.1.67) VCAM1; vascular cell adhesion molecule 1 VCL; vinculin.

Cell adhesion molecules and leukocyte transendothelial migration were among the top down-regulated pathways (Table 2, Fig. 8). Currently both pathways were found to correlate with recurrence after operation of stage I lung cancer in four large datasets, and thus seem to play a role in tumour aggressiveness [52]. Their role in chemoresistance is not clear but we already detected down-regulation of these pathways in mesothelioma versus normal parietal pleura. Here, CDH5 or vascular endothelial cadherin is a key protein controlling the endothelial barrier and its disruption by specific antibody both amplifies metastasis in normal mice and overcomes the genetic resistance in mice [53]. Moreover, CDH5 is a candidate tumour suppressor and low expression strongly correlated to worse survival in neuroblastomas [54]. Claudins are integral membrane proteins and components of tight junctions that serve as a physical barrier to prevent solutes and water from passing through between epithelial or endothelial cell sheets. CLDN1 (Fig. 3) and CLDN5 are down-regulated in many cancers [55] and also in mesotheliomas [56] and low expression of these claudins are associated with more aggressive prostate cancer [57]. Importantly, another tight junction gene, OCLN, occludin, was shown to be closely involved in resistance to apoptogenes as cisplatin and gamma irradiation, and re-expression of OCLN sensitized cancer cells to these agents [58]. VCL, vinculin encodes a cytoplasmic actin-binding protein regulating cell shape, integrin clustering, force generation, and strength of adhesion as well as regulating apoptosis. In colorectal cancer versus normal mucosa it was down-regulated and correlated to carcinogenesis, invasion, and metastasis of colorectal carcinoma [59]. Interestingly, the activation of vinculin sensitized melanoma to chemotherapy and increased adhesion of cells to extracellular matrix ligands, numbers of cell-matrix adhesions, and downstream signaling [60]. ESAM is specifically expressed at endothelial tight junctions and on platelets, and its down-regulation decreases neutrophil extravasation [61]. JAM2/JAMB inhibition decreases leukocyte infiltration [62].

Low CXCL12 and VCAM1 has been related to both cancer progression and improved prognosis in various cancers, but no clear relation to chemo-resistance has been reported. The down-regulation of leukocyte transendothelial micration and adhesion is thus connected to several mechanisms related to tumour aggressiveness and treatment resistance, although these have not been connected to either pemetrexed nor mesothelioma previously.

### Candidate resistance markers

In the gene expression profile of malignant mesothelioma versus normal pleura recently published, we found signatures related to multi-resistance [8]. If we recognize this signature as mesothelioma-specific, then the genes that are shared with the signature of acquired resistance should be of high importance. There were 150 genes common between the top 1500 tumour versus normal gene list and resistance profile (Supplemetary Excel Sheet 8) among them KRT7 was highly overexpressed (Fig. 3), and the most striking change in immunoreactivity was observed in its encoded protein cytokeratin 7 (CK7) with a 75% augmentation of the number of cells stained (Table 1, Fig. 4). Positivity for CK7 in more then 75% of cells is regularly observed in several types of carcinoma (www.proteinatlas.org), as well as mesotheliomas [63], but to our knowledge there has not been reported such a degree of augmentation of immunoreactivity for cytokeratins in the course of therapy resistance in carcinomas, nor in mesothelioma. Evidence for KRT7 in resistance specifically against anti-metabolites was shown by cellular response to 5-fluorouracil in 5-FU-resistant colon cancer cell lines during treatment and recovery where KRT7 was among the differentially overexpressed genes [64]. Moreover, when pre-treatment expression was compared with the expression in a primary resistant case, KRT7 was 3.2-fold overexpressed in the aggressive case (Fig. 5). Thus, this gene-protein couple could be explored further as a marker of tumour aggressiveness as well as for pemetrexed/carboplatin resistance. SDC1 and its encoded protein syndecan-1 (CD138) are overexpressed in mesothelioma and myeloma, but downregulation rather than overexpression has been linked to drug resistance [65]. Intriguingly, while SDC1 mRNA was overexpressed, the IHC showed down-regulation, thus pointing at a post-translatory degradation process involved (data not shown). Thus, only SDC1 mRNA expression could be further studied as a putative resistance marker.

Among the down-regulated, both transcripts of SPARCL1, encoding hevin, were changed more than 2-fold (Fig. 3). This gene has recently been assigned a tumour suppressor role as well as down-regulation in metastasis in pancreatic cancer [66]. Hevin IHC was chosen due to its primarily high gene expression and high down-regulation at resistance. It showed very strong staining in all pre-treatment tumour cells but only a slightly less strong staining post-resistance. Thus, SPARCL1 mRNA only could be a putative resistance marker. Of the remaining genes there is no established relation to pemetrexed or platinum resistance, but PMP22, SRPX are indicators of increased tumour progression and/or aggressivity in other cancers [67]. OSR1 is a transcription factor that regulates p53 in concert with other genes, and its overexpression activates p53 [68]. This may be very important but a direct role the progression of cancer has not been described. CDKN1B or its encoded protein p27 down-regulation is involved in several cancers, in mesothelioma low p27 protein expression correlated with short survival [69]. Low p27 also was predictive to survival of breast cancer after fluorouracil, cyclophosphamide, and methotrexate treatment [70]. Interestingly CDKN1B was down-regulated both in tumour versus normal and after resistance (Fig. 8). Recently, low p27 expression in lung cancer cells was correlated to pemetrexed resistance [71]. With this background, CDKN1B could also be explored as a putative resistance marker in mesothelioma. Selenoprotein 1 (SEPP1) was down-regulated as found in small-cell lung cancer [72].

### Protein versus mRNA expression

The following genes were all overexpressed in our previous study of mesotheliomas versus normal pleura, and were proposed as targets for treatment. We wanted to assess whether the protein expression of those genes was related to pemetrexed/carboplatin resistance.

TS and CHEK1 were discussed in the previous section. NQO1 is a detoxifying reductase, where lack of function in somatic cells is related to increased benzene oncogenesis and inactivating polymorphism has been related to dismal prognosis and predictive of treatment failure in breast cancer [73]. In tumour cell overexpression this is common and shown to induce proliferation in melanoma cells [74]. The encoded protein NQO1 was primarily highly expressed in cytoplasma, but low expressed in the nucleus. Although the gene was not differentially expressed at resistance, nuclear protein expression increased from 20 to 70%. Again, in our previous study, the NQO1 mRNA in the most aggressive and primary resistant case was 5.5-fold overexpressed (Fig. 5). Thus nuclear expression of this protein may as well be a marker of resistance. Previously we proposed this protein as a treatment target, as its overexpression is a prerequisite for the effect of the novel anticancer compound beta-Lapachone [75] that induce selective tumour apoptosis by an unknown mechanism, as well as radio-sensitisation *in vitro*. Moreover in cell lines beta-Lapachone is found to inhibit DNA polymerase alpha, DNA replication and TS activity, topoisomerase I, NFkappa-beta activity as well as induction of topoisomerase II alpha mediated DNA breaks [76]. Most of these pathways were overexpressed in mesothelioma, and thus this compound could be an interesting combination possibly with pemetrexed and platinum.

RAD21 gene expression was not differentially expressed and RAD21 protein expression was high in the primary biopsy where 90% of cells were positive, but there was a slight increase both in intensity and positive cell count after resistance. RAD21 is a critical gene in double-strand DNA repair and mitotic growth and gene overexpression was recently shown to be involved in invasion and metastasis in oral squamous cell carcinoma [77]. Silencing of RAD21 gene expression decreased cell growth and enhanced cytotoxicity of etoposide and bleomycin in human breast cancer cells [78].

AGGF1, a recently discovered potent angiogenic [79], and is implicated in damage response related to radiation defence, as ionizing radiation induces overexpression of AGGF1 in lymphoblastoid cells [80]. VG5Q, the protein encoded by this gene was highly overexpressed in more then 95% of tumour cells, and after progression virtually 100%. As it did not change significantly this gene/protein may not be directly involved in pemetrexed and platinum resistance but could be important for progression through stimulating neo-angiogenesis.

Finally, with the exception of membrane EMA, the routine immunohistochemical markers were not altered after resistance, and thus cannot serve as resistance markers or markers of changing biology (Table 1).

### Gene expression of the most sensitive versus the most aggressive case

In order to explore the possible gene expression differences of a sensitive case with more resistant case, the fold change of expression was compared with a case in our previous study with only six months survival [8]. The analysis revealed highly overexpressed DNA repair genes in the aggressive case (Fig. 5). Many-fold difference was detected in CHEK1, TOP2A and genes related to double-strand break repair. The TYMS gene was overexpressed only 1.5-fold. The aggressive case also had 8.25-fold increased HJURP and 11-fold increased CENPA. Recently, these genes were found to be involved in DNA double strand break repair and cell segregation as well as survival in breast cancer [81]. Interestingly, only CCNO/UNG2 was underexpressed in the aggressive tumour, and there is a hypothesis how this may contribute to antifolate resistance [82]. The glycosylase UNG2 initiates and is the rate-limiting factor for Base Excision Repair (BER) of uracil [83]. In nature, toxic uracil incorporation in DNA is removed by UNG2 and replaced by dTTP as should be. When dTTP is lacking due to antifolate treatment, the DNA break persist and the cell enters apoptosis. Overexpressed UNG2 and concordant BER could thus confer to more DNA breaks and tumour cell kill, while a low expression would not induce the same amount of DNA breaks and thus inhibit the effect of pemetrexed.

Among the top overexpressed genes in the primary resistant case versus the sensitive case, several very interesting genes were found (Fig. 5, fold-change within brackets in the text). The gene with the highest difference between aggressive and responsive tumour was the KRT14 (Keratin 14) (93-fold) where it recently was shown that the presence of Keratin14 positive progenitor airway epithelial cells in NSCLC predicted a poor prognosis, and this predictive value was strongest in smokers, in which it also correlated with metastasis [84]. OXTR, oxytocine receptor gene (80-fold) has also been detected in breast cancer cells with intrinsic and acquired resistance to doxorubicin [85]. Radio-resistance, early recurrence and metastasis are related to high CCNB1 (17.4-fold) expression in head and neck cancer as well as acquired radio-resistance, possibly through the activation of NFκB and other anti-apoptotic mechanisms [86], [87]. The KIF14 (Kinesin 14) (13-fold) is an oncogene related to several cancers and where mRNA overexpression is a negative prognostic factor in lung and breast cancer [88]. CEP55 (12-fold) encodes a centrosome-related gene where high expression was negative prognostic for head and neck cancer and its down-regulation inhibited migration and metastasis of cells [89]. SERPINB5 (9.8-fold) is strongly associated to breast cancer metastasis [90] and negative prognostic in pancreatic cancer [91]. BUB1 (9.6-fold) and BUB1B (5.7-fold) are involved in the spindle assembly checkpoint, and overexpression of BUB1B significantly correlated with higher histological grade, advanced pathological stage, and high cell proliferation in bladder cancer and predicted tumour recurrence and disease progression [92]. BUB1 is also a possible negative prognostic factor in mesothelioma [93]. Survivin, encoded by BIRC5 (7.6-fold) has, besides its anti-apoptotic function, a role in microtubule dynamics and control bipolar spindle formation [94]. Survivin is overexpressed in many human cancers, associated with resistance to chemotherapy or radiation therapy, and linked to poor prognosis, also in mesothelioma [95], [96]. Importantly, survivin also seems to control Ran, encoded by RAN (2-fold). Ran is a small GTPase regulator of mitotic spindle formation and is overexpressed in human cancer as compared with normal tissues. Gene silencing of RAN induces aberrant mitotic spindle formation, mitochondrial dysfunction, and apoptosis [97]. CDC2 (9.4-fold) is a spindle checkpoint gene and overexpression is a negative prognostic factor in several tumours [98] as well as a putative treatment target in gliomas [99]. Its overexpression was also verified in a cohort of 84 mesotheliomas [50]. LRP8 (8-fold) is overexpressed in lung cancer and involved in lung tumorigenesis [100]. CDC20 (7.5-fold) appears to act as a regulatory protein in the cell cycle and is required for two microtubule-dependent processes, nuclear movement prior to anaphase and chromosome separation. A signature of genes including CDC20, CCNB1, CDC2, CDKN3, MAD2L1, PRC1 and RRM2, were prognostic for 5-year survival in over 400 lung cancer cases [101], and interestingly, CDC20, CDC2, CCNB1 were also highly overexpressed in the aggressive case. E2F7 (7.4-fold) is implicated in damage response to DNA damaging agents [102]. CCNB2 (7.4-fold) another cyclin overexpressed in several cancers, was interestingly proposed as a serum marker for various cancers as serum CCNB2 mRNA was significantly elevated in patients versus benign diseases or normal [103]. AURKA (6.8-fold) overexpression leads to centrosome amplification, genetic instability and transformation, as well as cisplatin resistance. Its activation of the NFkB pathway has been proposed as an important mechanism [104]. AURKA is overexpressed in several cancers, and has been associated with shorter survival in mesotheliomas [105] and small molecule inhibitors of AURKA are currently in phase II trials [106]. MKI67 (6.2-fold) encodes an antigen identified by monoclonal antibody Ki-67, a nuclear protein and proliferation marker that is a negative prognostic for pleural mesothelima [69].

**Figure 9:**
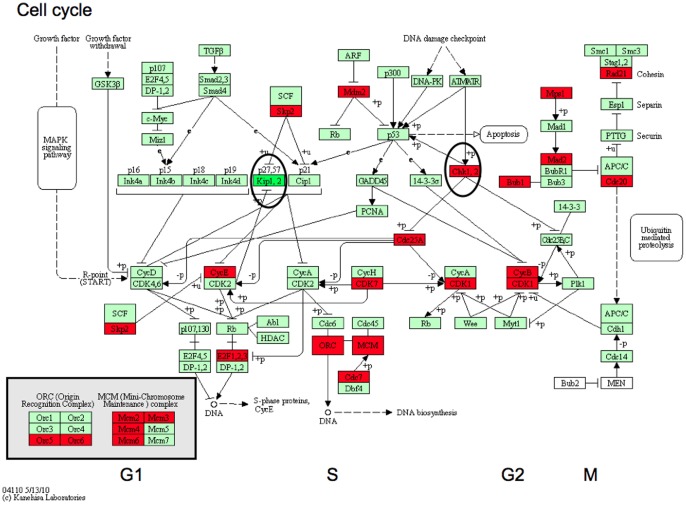
Cell cycle changes tumour versus normal and acquired resistance. Cell cycle was one of the KEGG pathways with most de-regulated genes both in tumour versus normal (23 genes- red correspond to overexpressed, dark green correspond to down-regulated) involved in all phases of the cell cycle (G1-S-G2-M). At acquired resistance, only CDKN1B (in oval) was down-regulated and CHEK2 was overexpressed (in oval). Abbrevations: CDKN1B; cyclin-dependent kinase inhibitor 1B (p27, Kip1) CHEK2; checkpoint kinase 2.

Among those top overexpressed genes, 23 are involved in cell cycle (Fig. 9), cytokinesis, and spindle formation, and several are known to be negative prognostic factors in other cancers. The grave difference in gene expression between these two phenotypically different cases in the primary situation indicate that some important systems are more deregulated in the aggressive case. However, most of the genes that changed in acquired resistance were different.

### Serum biomarkers

Neither the MSLN gene nor its encoded protein changed in tumour at resistance and tumour growth (Table 1) but serum mesothelin was reduced at response and increased at resistance (Fig. 6). While serum mesothelin is a valuble mesothelioma marker for diagnosis and disease progression, we have previously shown that increased mesothelin expression in tumour was not associated to shorter survival, rather the opposite [15], lending notion to the theory of tumour burden as the main source of SMRP change and not tumour aggressiveness. Similarly, serum CA125 was positively associated to clinical and radiological course, but its encoding gene MUC16 did not change in the tumour, also indicating that tumour burden and not differential gene expression in tumour increases serum levels. Neither CYFRA21-1 in serum nor its encoding KRT19 gene in the tumour changed significantly.

## Conclusion

More than half of mesothelioma patients do not respond to the standard treatment of pemetrexed and cisplatin and acquired resistance in responders is almost obligate. Thus, biological information leading to tailored treatment is clearly warranted.

We present here for the first time, an example of *in vivo* resistance gene profile of pemetrexed-carboplatin treatment in mesothelioma. At resistance the TS protein, the main target of pemetrexed increased. DNA damage response, repair, elongation and telomere extension, related directly and indirectly to platinum resistance were overexpressed, such as the CHEK2 gene, the CHK1 protein, the POLD1, POLA2, LIG3, FANCD2 and finally the novel DNA repair genes PRPF19 and RECQ5, not previously described in mesothelioma. Down-regulation of leukocyte transendothelial migration and cell adhesion genes were overrepresented and are novel pathways involved in resistance and tumour aggressiveness. Overexpression of KRT7 along with its encoded protein CK7 and TYMP with its encoded highly pro-angiogenic thymidine phosphorylase protein as well as down-regulated SPARCL1 and CDKN1B are proposed resistance markers. Comparison of the primary expression of the sensitive versus a primary resistant case showed multi-fold overexpressed DNA repair, cell cycle, cytokinesis, and spindle formation in the latter. Serum CA125 and SMRP reflected the clinical and radiological course and probably tumour burden.

The present analysis and previous microarray study is to our knowledge the only genome-wide profiling study on mesothelioma patients receiving chemotherapy as the main treatment, which is by far the largest group of patients treated in clinical practice [9]. Thus, these findings should be confirmed in a larger patient cohort with profiling of biopsies before treatment and after treatment failure. Such a study is strongly recommended in order to achieve a deeper understanding of mesothelioma resistance mechanisms *in vivo* and identify the markers that may guide treatment decisions to improve and personalize treatment in this patient group.

## Supporting Information

Figure S1 **Loading plots and Principal Component Analysis** (**PCA**)**.** The coloured spots in the loading plots above represent differentially expressed genes where red spots in the middle represent Affymetrix and blue represent Illumina, green represent low variance genes overlapping differentially expressed genes between the platforms. The PCA score-plots of the gene expression of the same RNA from the same samples on Affymetrix and Illumina platforms below are virtually identical (see case IDs), where red are tumour and black are parietal pleura samples.
(TIFF)

Figure S2 **Venn diagram of up-** (**red**) **and down-regulated** (**green**) **genes of mesothelioma tumour versus normal parietal pleura.** An overlap between the Affymetrix and Illumina platforms of 65% is seen.
(TIFF)

Figure S3 **Silencing of NT5C and pemetrexed treatment.** Cell cycle distribution of NT5C silenced (+) or negative siRNA control (–) in malignant mesothelioma cells, after 48 hours of pemetrexed treatment. The percentages represent the amount of live cells in control and pemetrexed treated cells. Levels of significance: *  = P<0.05, **  = P<0.01, ***  = P<0.001, ****  = P<0.0001.
(TIFF)

Table S1 **siRNA specific for NT5C** (**Sigma-Aldrich**)**.**
(DOCX)

Table S2 **Sheet 1–8. Sheet 1–3.** The top 1500 ranked list and GO entities of differentially expressed genes showed an overexpression of genes involved in mitosis, cell cycle checkpoint and DNA repair and down-regulated multicellular organismal development, inflammatory response and vasculogenesis among others. **Sheet 4–7.** High quality RNA was successfully extracted from the post-resistance tumour samples and analysed with the Illumina platform as described. The acquired resistance analysis revealed 241 overexpressed and 289 down-regulated genes in the post-resistance samples (Sheet 4 and 5). There were 23 significantly overexpressed and 65 down-regulated GO-terms (Sheet 6 and 7, p<0.05). **Sheet 8.** Acquired resistance and tumour profile. Common gene list.
(XLS)
