# Supplementary figures and images for: Molecular Resistance Fingerprint of Pemetrexed and Platinum in a Long-Term Survivor of Mesothelioma
Source: PLoS One. 2012 Aug 8;7(8):e40521. doi: 10.1371/journal.pone.0040521 (PMC3414492; doi:10.1371/journal.pone.0040521)

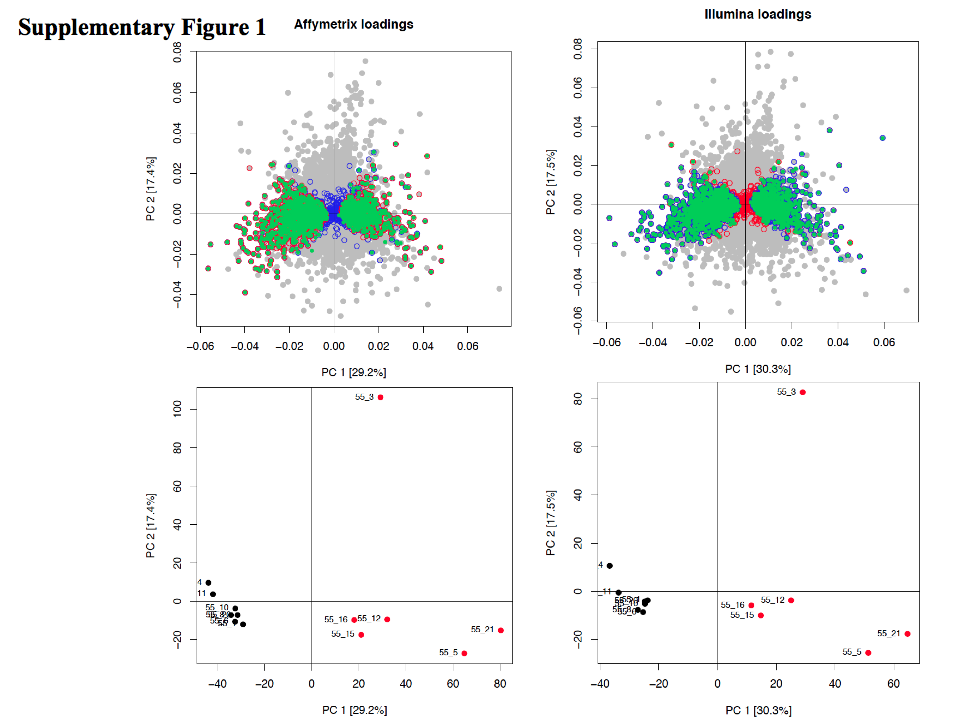

Supplement: Figure S1 — Loading plots and Principal Component Analysis (PCA). The coloured spots in the loading plots above represent differentially expressed genes where red spots in the middle represent Affymetrix and blue represent Illumina, green represent low variance genes overlapping differentially expressed genes between the platforms. The PCA score-plots of the gene expression of the same RNA from the same samples on Affymetrix and Illumina platforms below are virtually identical (see case IDs), where red are tumour and black are parietal pleura samples. (TIFF) [file pone.0040521.s001.tiff]

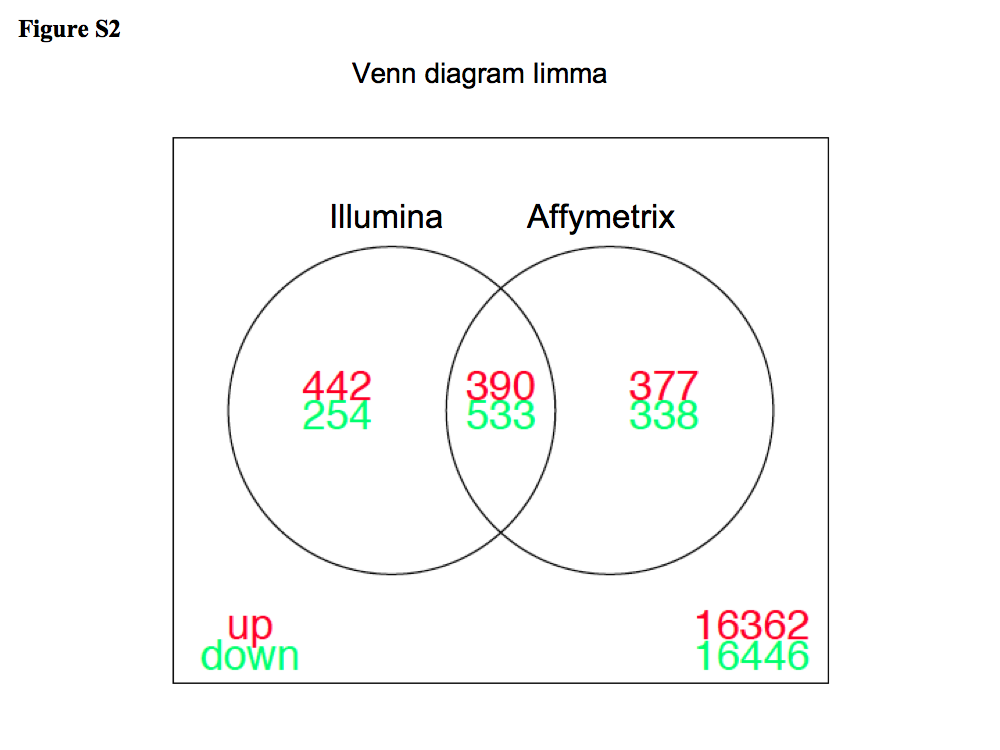

Supplement: Figure S2 — Venn diagram of up- (red) and down-regulated (green) genes of mesothelioma tumour versus normal parietal pleura. An overlap between the Affymetrix and Illumina platforms of 65% is seen. (TIFF) [file pone.0040521.s002.tiff]

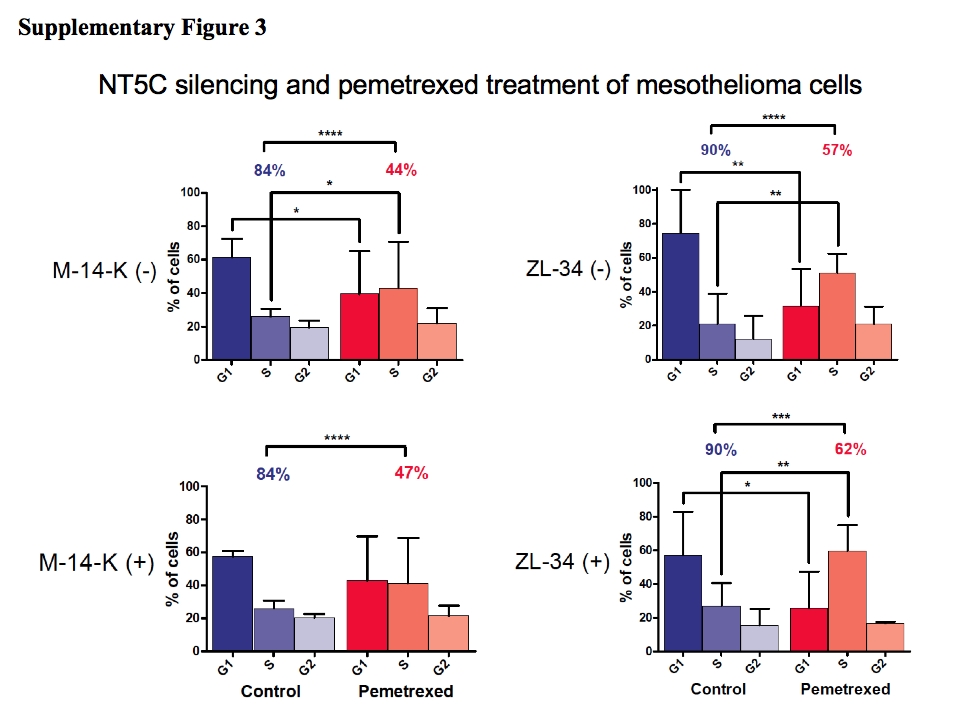

Supplement: Figure S3 — Silencing of NT5C and pemetrexed treatment. Cell cycle distribution of NT5C silenced (+) or negative siRNA control (–) in malignant mesothelioma cells, after 48 hours of pemetrexed treatment. The percentages represent the amount of live cells in control and pemetrexed treated cells. Levels of significance: * = P<0.05, ** = P<0.01, *** = P<0.001, **** = P<0.0001. (TIFF) [file pone.0040521.s003.tiff]
