# Supplementary material for: Molecular Resistance Fingerprint of Pemetrexed and Platinum in a Long-Term Survivor of Mesothelioma
Source: PLoS One. 2012 Aug 8;7(8):e40521. doi: 10.1371/journal.pone.0040521 (PMC3414492; doi:10.1371/journal.pone.0040521)
Supplement: Table S1 — siRNA specific for NT5C (Sigma-Aldrich). (DOCX) [file pone.0040521.s004.docx]

**Supplementary Table 1.**

siRNA construct sequence (sense 5`-3`) for NT5C silencing and primer sequences (5` to 3` orientation) for qRT-PCR.

| NT5C siRNA, MISSION® siRNA from Sigma-Aldrich: GAAUUAUCCUGACAAGGGA and UCCCUUGUCAGGAUAAUUC |
| --- |
| NT5C forward: GGA CAC GCA GGT CTT CAT CT  NT5C reverse: GCC TCG ACC TGT GTC CTT GT |
| GAPDH forward: ACA TCA TCC CTG CCT CTA CTG G  GAPDH reverse: AGT GGG TGT CGC TGT TGA AGT C |

.
